# Molecular epidemiology of *Toxoplasma gondii* in impala (*Aepyceros melampus*) from the Greater Kruger in South Africa: Detection of the Africa 4 lineage

**DOI:** 10.1371/journal.pntd.0014475

**Published:** 2026-07-09

**Authors:** Madeli de Bruin, Shanzelle Rabé, Okuhle N. Sekujika, Karine Passebosc-Faure, Virginie Rougeron, Almero D. Bosch, Pierre Dorny, Isabelle Villena, Kenneth Hammond-Aryee, Paul van Helden, Monika Esser, Aurélien Mercier, Luis Neves, Franck Prugnolle, Darshana Morar-Leather

**Affiliations:** 1 Cyst-forming Protozoan Parasite Research Group, Department of Veterinary Tropical Diseases, Faculty of Veterinary Sciences, University of Pretoria, Pretoria, South Africa; 2 Inserm U1094, IRD, Univ. Limoges, CHU Limoges, EpiMaCT - Epidemiology of chronic diseases in tropical zone, Institute of Epidemiology and Global Health – Michel Dumas, OmegaHealth, Limoges, France; 3 National Reference Centre (NRC) for Toxoplasmosis, Limoges University Hospital, Limoges, France; 4 IRL REHABS, International Research Laboratory REHABS, CNRS, Nelson Mandela University, George, South Africa; 5 Sustainability Research Unit, George Campus, Nelson Mandela University, George, South Africa; 6 Timbavati Private Nature Reserve Abattoir, Hoedspruit, Mpumalanga, South Africa; 7 Department of Biomedical Sciences, Institute of Tropical Medicine, Antwerp, Belgium; 8 University of Reims Champagne-Ardenne, ESCAPE, Reims, France; 9 Reims University Hospital Centre, National Toxoplasmosis Reference Centre, Toxoplasma Biological Resource Centre, Reims, France; 10 South African Medical Research Council Centre for Tuberculosis Research, Division of Molecular Biology and Human Genetics, Faculty of Medicine and Health Sciences, Stellenbosch University, Cape Town, South Africa; 11 Biotechnology Centre, Eduardo Mondlane University, Maputo, Mozambique; Advanced Centre for Chronic and Rare Diseases, INDIA

## Abstract

**Background:**

*Toxoplasma gondii* is an apicomplexan parasite that causes toxoplasmosis, a widespread zoonotic disease. Despite the clinical significance of this zoonotic parasite, little is known regarding its prevalence in South Africa, particularly in wildlife. Considering the possible presence of the parasite in wildlife species and the popularity of South African game meat, in a ‘One Health context’, the consumption of undercooked game meat by people could represent a public health issue. The objective of this study was to determine the prevalence of *T. gondii* and any genotypes in impala from the Greater Kruger region destined for game meat.

**Methods:**

Serum and seven different tissue samples were collected from 138 impala (*Aepyceros melampus*) from the Timbavati Private Nature Reserve in South Africa. The seroprevalence of *T. gondii* was determined using the Modified Agglutination Test (MAT). The presence of *T. gondii* DNA within the impala tissues and possible tissue tropism were determined using a quantitative PCR (qPCR). For strong qPCR-positive samples, *T. gondii* DNA was genotyped using a panel of 15 microsatellite markers.

**Results:**

The seroprevalence was determined to be 8.7%. The qPCR identified *T. gondii* DNA in at least one tissue type of 7.2% of the impala. The *T. gondii* DNA was detected in the brain and tongue samples from two impala respectively, and were genotyped as belonging to the Africa 4 lineage. To place the two genotypes identified in this study within the broader context of the genetic diversity of *T. gondii* in Africa, a genetic tree was constructed using all African strains genotyped with 15 microsatellite markers.

**Conclusion:**

These results shed light on serological versus molecular techniques in determining infection of *T. gondii* in impala, and also point to possible tissue tropism during infection. The results identify Africa 4 strains circulating in South African wildlife intended for human consumption, and the importance of genotype and phenotype characterisation to assess the potential public health risks.

## Author summary

This study investigated *Toxoplasma gondii* infection in impala by detecting anti–*T. gondii* IgG antibodies in serum and *T. gondii* DNA in tissue samples. The main goal was to determine *T. gondii* infection in impala and identify the specific parasite genotypes circulating in this species. Understanding the extent of *T. gondii* infection in the impala population from Timbavati is critical, particularly because these animals are regularly culled and consumed as meat in both rural and urban settings. Identifying which tissues harbour *T. gondii* cysts further clarifies the potential pathways for zoonotic transmission. The detection of the Africa 4 genotype in impala provides new insight into *T. gondii* strain diversity in wildlife and highlights the need for future studies examining phenotype and pathogenicity to better evaluate risks to public health.

## Introduction

*Toxoplasma gondii* is a zoonotic, intracellular protozoan parasite, transmitted between felid definitive hosts and warm-blooded intermediate hosts [1]. This parasite is distributed globally and causes toxoplasmosis in animals and humans, which is generally asymptomatic. However, it can cause severe symptoms such as fever, headaches, seizures, eye damage, nausea and poor coordination in immunocompromised individuals [2]. During pregnancy, it can cause malformation or even spontaneous abortions if transmitted to the fetus [3]. Despite the significance of this zoonotic parasite for both veterinary and public health, there is very limited research on *T. gondii* in relation to the wild African environment. The risk of contracting *T. gondii* is significant in the African population, given the high prevalence of HIV infection, and the fact that immunocompromised patients contract a potentially fatal neurological opportunistic infection with *T. gondii* [4]. More research is needed in terms of both clinical significance and the transmission between wildlife, domestic animals and humans at wildlife-domestic interface areas in rural settings [5].

Research has historically focused on seroprevalence in human and domestic species, with limited attention paid to the toxoplasmosis transmitted by consumption of meat from wildlife. Bakal, Karstad [6] found a seroprevalence of 80% in impala (*Aepyceros melampus*) in Kenya using the Sabin–Feldman dye test. A study by Bokaba, Dermauw [7] in South Africa showed a 5.2% seroprevalence in impala using the Latex Agglutination Test (LAT). Different wild herbivore species also yielded varying results when compared to those of the impala. Hove and Mukaratirwa [8] used the Modified Agglutination Test (MAT) on a variety of wildlife species in Zimbabwe, and found a seroprevalence of 10% in giraffe (*Giraffa camelopardalis*), 20% in Greater kudu (*Tragelaphus strepsiceros*), 90% in nyala (*Tragelaphus angasii*), and 57% in bushbuck (*Tragelaphus sylvaticus*). Lukášová, Halajian [9] made use of an ELISA and found the seroprevalence of *T. gondii* in Greater kudu to be 8%. It should also be noted that the sample sizes in these studies are relatively small, with the exception of the study by Bokaba, Dermauw [7], and that the serological tests have different sensitivities and specificities.

These types of studies remain relevant, as game or bush meat is consumed on a regular basis in many parts of Africa [10]. This is the case in rural communities in and around the Kruger National Park (KNP) in South Africa. Although these meats are sometimes consumed undercooked, they are also often salted and cured and sold as biltong in shops, mostly for tourists, but also for local consumption. Another common game meat is springbok carpaccio which is served raw and is considered a delicacy. *T. gondii* tissue tropism has been investigated in different host species [11–14]. Although there seem to be similarities between species, no studies have been conducted to specifically determine which tissues in impala, a species widely consumed in South Africa, may be predilection sites for *T. gondii* and therefore pose a significant potential risk of transmission to humans.

In addition to detecting the parasite, genetically characterising *T. gondii* strains is important because strains can vary in virulence, clinical impact, and epidemiological significance depending on their genotype [15,16]. Certain strains are known to be more virulent, leading to more severe outcomes in humans or animals [16]. A discriminatory tool that is popular and often used for the typing of *T. gondii* strains is microsatellite (MS) markers [17]. In Africa, various strains have been described in patients with ocular (Type I), acute (Type I, II, III, atypical, Africa 1, mixed), and congenital toxoplasmosis (Type I, II, atypical, recombinant), as well as in immunocompromised patients (Type I, II, III, Africa 1, Africa 2, mixed, recombinant) [15,18], but data linking genetic and clinical diversity remain extremely rare.

The main aim of this study was to determine the seroprevalence and genetically characterise *T. gondii* infection in the impala population from the Timbavati Private Nature Reserve (TPNR), a reserve adjacent to the Kruger National Park in South Africa, to evaluate its potential zoonotic risk and public health impact of infected impala meat.

## Materials and methods

### Ethics statement

This study received Research and Animal Ethics approvals from the University of Pretoria’s Research and Animal Ethics Committees (REC-015–23), respectively. In addition, Section 20 approval and amendments (reference numbers: 12/11/1/1/8 (2998PM), (5001PM), (5115PM)) were obtained from the Department of Agriculture, Land Reform and Rural Development. All samples were collected, transported, processed, and stored according to the Section 20 permit (reference number: 12/11/1/1/6/5068/(HP).

### Ethics approval

Approval to collect samples from the Timbavati abattoir was provided by the Mpumalanga Tourism and Parks Agency (MTPA) and the Timbavati Private Nature Reserve (TPNR). Permission to use the laboratory facilities at the Hans Hoheisen Wildlife Research Station (HHWRS) was also granted. Movement permits were also issued for extracted DNA transported from HHWRS to the Research and Training laboratories of the Department of Veterinary Tropical Diseases at the Onderstepoort campus of the University of Pretoria. A veterinary import permit and dispensation letter (Permit number: 13/1/1/30/2/0–202308003967) were obtained for the importation of *Toxoplasma gondii* DNA (FOU, ME49, and NED strains) from the Université de Limoges in France.

### Study site

**Fig 1:**
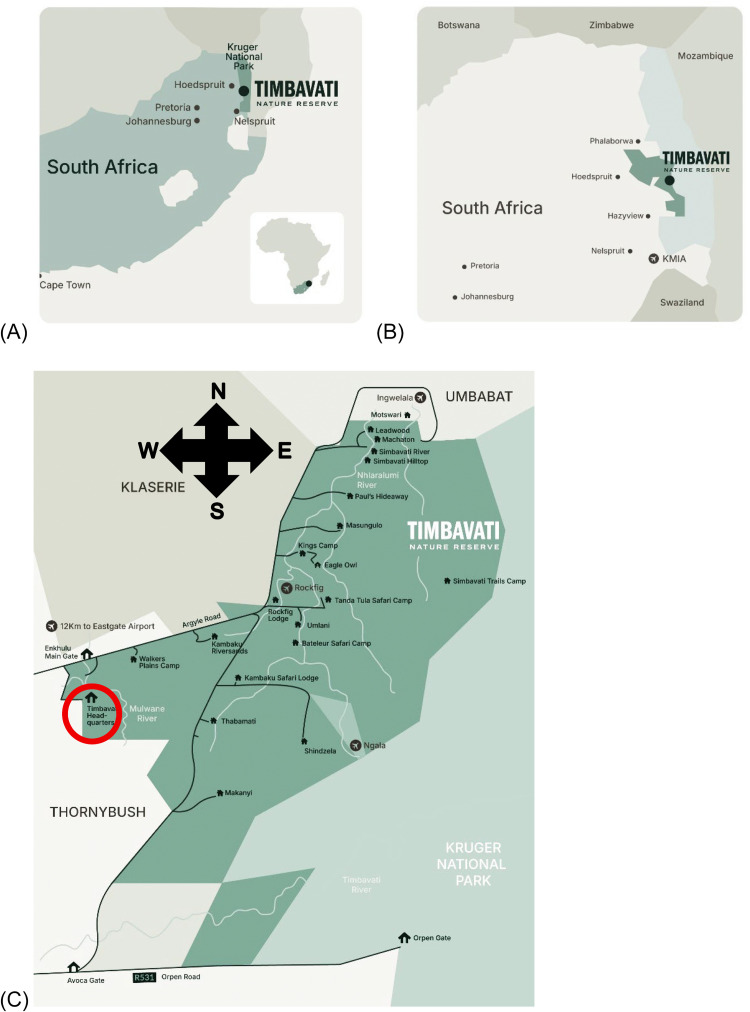
Timbavati Private Nature Reserve. **(A)** A map showing the location of the TPNR in South Africa. **(B)** The TPNR is located within the Limpopo province. **(C)** The TPNR borders the western boundary of the Kruger National Park. The Timbavati HQ (red circle) indicates where the abattoir is located [19]. Image credit: https://www.timbavati.co.za/getting-here.

Samples for this study were obtained from the Timbavati abattoir, located within the TPNR in the Limpopo province, South Africa (Fig 1). The TPNR is over 53 000 hectares and is located on the western boundary of the KNP.

Timbavati is part of the Greater Kruger conservation area and features a savanna ecosystem that supports a rich diversity of wildlife, particularly ungulates. In the TPNR, animals roam freely between Timbavati and the Kruger National Park because there are no fences separating them. Impala are regularly culled at Timbavati for population control and the carcasses are processed at the abattoir. Adult impala (*Aepyceros melampus*) samples were collected for over six months, from November 2023 until April 2024.

The sample size for the project was calculated to be 138, according to the sample calculation for estimation of pathogen prevalence, using simple random sampling [20].


$$\begin{array}{c} \text{n }=\;\frac{{\text{1}.\text{9}\text{6}}^{\text{2}}P_{exp}(\text{1}-P_{exp})}{d^{\text{2}}}\;=\text{ 138} \end{array}$$

With n being the required sample size, P_exp_ is the expected prevalence, and d is the desired absolute precision. Therefore, for a 10% expected prevalence and 5% desired precision, at least 138 impala were needed.

### Sample collection

Blood from the impala was collected in serum tubes from the jugular vein. The collected blood was kept at 4°C. Serum was obtained from the collected blood via centrifugation at 2000 x g and was stored at -20°C until used. The tongue, heart, diaphragm, triceps brachii, liver, and spleen tissue samples were taken aseptically using an 8 mm biopsy punch. The brain sample was obtained by first incising the skin using a scalpel, and then making a 3 cm hole in the skull through which a 1x1x1 cm piece of brain could be removed. All tissue samples were preserved in absolute ethanol in sample containers, and stored at 4°C until DNA extraction took place. A total of 138 impala were sampled, adding up to a total of 138 serum samples and 966 tissue samples (138x7 = 966).

### Modified Agglutination Test (MAT)

MAT [21] was used to determine seroprevalence with *T. gondii* by detecting IgG antibodies in the impala serum samples. The protocol makes use of dithiothreitol (DTT), which acts as a reducing agent to disrupt the disulphide bonds of the IgM antibodies. The antigen used consisted of tachyzoites that had been formolised from the *T. gondii* RH strain. Toxoplasma antigen was provided free of charge by National Reference Center on Toxoplasmosis (Reims, France). Serological controls were fresh blood from seronegative and experimentally infected seropositive Swiss mice (*Mus musculus*). These control sera represent a quality control for each series of serological tests.

The antigen was diluted in borate-albumin-buffered saline (BABS) to a 1/17 solution, which was kept at 4°C. The BABS buffer (pH 8.95, 4°C) was made manually using: 3.092 g boric acid, 4 g bovine albumin, 1 g sodium azide, 7.012 g sodium chloride, 24 mL sodium hydroxide (1 N), and made up to 1000 mL with distilled water [22]. DTT was diluted 1:50 with PBS buffer (pH 7.2). This DTT/PBS solution was diluted 1:2 with PBS and stored at 4°C. The MAT was performed using a serial dilution (1:20, 1:40. 1:100 and 1:800) with 1:20 as the positive threshold, as previously described by Bolais, Vignoles [23].

MAT was performed using 96-well round-bottomed microplates. In the first row, 95 µL of the DTT/PBS was added to each well. In the second, row 25 µL of DTT/PBS was added to every well. In the third row, 37.5 µL DTT/PBS was added to every well. In the fourth row, 175 µL DTT/PBS was added to every well. Subsequently, the serum sample (5 µL) was added to each well in the first row. Positive (*T. gondii*-infected mice) and negative (*T. gondii*-free mice) control sera were used in the MAT assay.

The dilution was performed as follows: 50 µL was discarded from the wells in the first row, 25 µL was transferred from the first to the second row, 25 µL was transferred from the second to the third row, 12.5 µL was discarded from the third row, and 25 µL was transferred from the third to the fourth row, and finally 175 µL was discarded from the fourth row. After the dilution series, 25 µL of the BABS-antigen buffer was added to all the wells. The plate was incubated at room temperature in the dark, for 12–24 hours, before the results were viewed. A hazy cloudy white layer was indicative of a positive case, whereas a white pellet at the bottom of the well indicated a negative result. The positive and negative controls were checked to confirm the reliability of the results.

### DNA extraction

The different tissue samples were cut into smaller pieces (50 mg) using a blade and transferred to Eppendorf tubes along with 300 μL lysis buffer (pH 8, 10 mM Tris-base, 100 mM EDTA, 2% SDS), and 10 μL Proteinase K. The samples were incubated overnight (56°C). After an overnight incubation, the samples were vortexed, and 400 μL of 4 M sodium acetate was added. Next, the samples were vortexed and incubated at -20°C for 30 minutes. The samples were then centrifuged for 20 minutes at 13 000 x g. The supernatant was then transferred to a new tube, and the pellet was discarded. Absolute ethanol (600 μL) was added to the supernatant, and the tube was gently inverted. The samples were then centrifuged for 10 minutes at 13 000 x g, and the supernatant was discarded. The pellet was washed with 600 μL of 70% ethanol, followed by another centrifugation step of 20 minutes at 13 000 x g. This step was repeated. The ethanol was discarded, and the pellet was dried. The extracted DNA was resuspended in 200 μL TE buffer (pH 8, 10 mM Tris-base, 0.1 mM EDTA), and stored for long-term use (-20°C). Spectrophotometry was done on a few DNA samples to confirm the purity and concentration of the DNA. Good purity was defined as an A260/A280 ratio of 1.8 and an A260/A230 ratio of 2.

### Detection of *T. gondii* by qPCR

The qPCR protocol targeted an 81-base pair (bp) fragment of the non-coding 529-bp repeat element (RE) that occurs throughout the *T. gondii* genome [24]. The primer sequences (Forward: 5′-CAC AGA AGG GAC AGA AGT CGA A-3′ and Reverse: 5′-CAG TCC TGA TAT CTC TCC TCC AAG A-3′) and probe sequence (FAM-5′-CTA CAG ACG CGA TGC C-3′-quencher) were used as previously described [24], except for using the BMN-Q535 3’-quencher for the probe.

The qPCR method [24] was adjusted using the TaqMan Universal PCR Master Mix user guide. The qPCR reaction master mix consisted of 1X Taqman Universal Master Mix, 0.5 μM of both the forward and reverse primers, 0.1 μM of the probe, DNA template (5 µL), and distilled water up to a final total volume of 25 μL. The qPCR-positive control was the *T. gondii* FOU strain (Africa 1 lineage). The reactions were run on the StepOne Real-Time PCR System. The qPCR protocol consisted of a holding stage of 50°C for 2 minutes and 95°C for 10 minutes, followed by 45 cycles of 95°C for 15 seconds and 62°C for 1 minute.

In this study, a FOU strain (Africa I), with a concentration of 4 tachyzoites per microlitre and a corresponding Ct value of 25, was used as the reference point. *T. gondii* DNA detected above 25 cycles (Ct values) and below 42 cycles was considered a weak positive, and detection below and equal to 25 cycles was considered a strong positive. This classification of positivity was merely qualitative (descriptive) in this study. This range was determined by performing a dilution series (in duplicate) of the FOU strain (4 tachyzoites/uL): undiluted, 1/10, 1/100, 1/1000 and 1/10 000. Where the undiluted sample had an average Ct value of 25, and the last dilution 1/10 000 had an average CT value of 42,60. qPCRs were repeated in duplicate on positive samples (samples with Ct values below 42) to confirm the results. Any samples with a Ct value above 42 or no Ct values (no amplification curve) were regarded as negative.

### Statistical analysis

A descriptive statistical analysis was conducted. Seroprevalence and molecular prevalence data are presented in percentages with 95% confidence interval (CI). All statistical procedures were run using Rstudio using the ‘binom’ package and the Wilson method [25].

### Genotyping of microsatellite markers: multiplex and simplex PCR

A panel of 15 MS markers has been previously developed for *T. gondii,* enabling the differentiation of lineages: eight lineage typing markers (TUB2, W35, TgM-A, B18, B17, M33, IV.1, XI.1) and seven fingerprinting markers (M48, M102, N60, N82, AA, N61, N83) [17,26]. The lineage typing markers are semi-polymorphic and distinguish between lineages, while the highly polymorphic fingerprinting markers can differentiate between strains (genotypes) belonging to a single lineage [17,27]. In this study, markers (W35, TgM-A, B17, IV.1) were labelled with 5’ fluorescent dye VIC. Whereas, markers (M102, N60 and AA) were labelled with 5’ fluorescent dye NED. All other markers (TUB2, B18, M33, XI.1, M48, N61, N82, N83) were labelled with 5’ fluorescent dye FAM.

qPCR-positive samples with a Ct value lower than 32 were genotyped by using 15 MS markers [17,26,27]. Multiplex PCRs were done to amplify the MS markers as previously described [26]. The *T. gondii* reference strains: FOU (Africa 1), ME49 (Type II), NED (Type III) were used as positive controls for allele size calibration. Distilled water (no DNA template) was used as the negative control. The multiplex master mix (total volume of 25 μL) consisted of 1X QIAGEN 2X Multiplex PCR Kit, 0.2 μM of the labelled forward and unlabelled reverse primers, 5 μL of DNA template, and distilled water. The multiplex PCR protocol had a denaturation step of 95°C for 15 minutes, followed by 35 cycles of a denaturation step of 94°C for 30 seconds, an annealing step of 61°C for 3 minutes, and an elongation step of 72°C for 30 seconds. Lastly, there was a final elongation step of 60°C for 30 minutes. When genotyping (fragment scoring) using the multiplex PCR method was incomplete (<15 MS), simplex PCRs were performed for each of the missing markers or to confirm the allele of a marker that initially struggled to amplify. For simplex PCRs, the annealing temperature(s) specific to each MS marker’s primer pair were used, as previously described [17].

The simplex PCR products (2 μL), along with 1 μL of 6X DNA loading Dye, were run on a 2% agarose gel, at 100 V for 50 minutes, in order to visualise (with ethidium bromide) whether amplification of the MS markers was successful. The expected lengths of the MS markers were verified by using a 100 bp DNA ladder. The gel served as a form of quality control (QC).

### Genotyping of MS markers: capillary electrophoresis and fragment scoring

The amplified MS products were prepared for fragment analysis. The PCR product or diluted PCR products (1 µL) were added to a 9 µL master mix consisting of GeneScan 500 LIZ Size Standard and Hi-Di Formamide (6 μL:250 μL ratio). The samples were denatured by placing the plate in the SimpliAmp Thermal Cycler at 96°C for 4 minutes. The PCR products were analysed with capillary electrophoresis (GeneScan) using the ABI3500xL Genetic analyser. The services of the DNA Sanger Sequencing facility at the Faculty of Natural and Agricultural Sciences (University of Pretoria) were used for GeneScan analysis. Allele size calling (scoring) was determined using the MS Analysis application (Applied Biosystems, Thermo Fisher Scientific).

The allele sizes obtained for the four markers (W35, TgM-A, B17, and IV.1), which were labelled with the fluorescent dye 5’ VIC rather than the 5’ HEX dye used in the original genotyping protocol [17], were converted according to the procedure described in [26], using the corresponding marker values from the three reference control strains (FOU, ME49, and NED).

To visualise the genetic relationships among *T. gondii* genotypes from various African countries, a neighbour-joining tree was constructed based on Bruvo’s genetic distances calculated from the 15 microsatellite markers. This analysis included the two genotypes identified in the present study, together with all African strains previously genotyped using the same 15 microsatellite markers (S3 Table). The *T. gondii* microsatellite database (S3 Table) was prepared for Rstudio using GenAlEx 6.5 [28]. The neighbour-joining tree was drawn using the ‘poppr’ and ‘ape’ packages in Rstudio [29,30]. The tree was edited and finalised in ITOL [31].

## Results

### Serology

A *T. gondii* seroprevalence of 8.7% (n = 12/138, 95% CI: 5.0 - 14.6) was observed using MAT. This means that 12 impala tested positive for anti-*T. gondii* antibodies (Table 1). Only two impala (F18 and F29) showed strong seropositivity at a high dilution (1/800 dilution) (S1 Table). One impala (F24) showed a seropositivity to the 1/400 dilution, and two impala (N36 and D6) were seropositive until the 1/100 dilution (S1 Table). Seven impala (N5, N59, N66, D3, F15, F33 and M33) were seropositive until the 1/40 dilution (S1 Table).

**Table 1:** Comparison between positive MAT and qPCR samples. Results of MAT, compared with qPCR results, of samples which were either seropositive but qPCR negative, seronegative but qPCR-positive, or positive in both tests.

Sample	MAT	qPCR						
		Tongue	Heart	Diaphragm	Triceps	Liver	Spleen	Brain
N5	+	–	–	–	–	–	–	–
N10	–	–	–	–	+	–	–	–
N36	+	–	–	–	–	–	–	–
N49	–	–	+	+	+	–	–	–
N50	–	–	–	+	–	–	–	–
N59	+	–	–	–	–	–	–	+
N61	–	–	–	–	+	–	–	–
N66	+	–	–	–	–	–	–	–
D3	+	–	–	–	–	–	–	–
D6	+	–	–	–	–	–	–	–
F15	+	–	–	–	–	–	–	–
F18	+	–	–	–	–	–	–	+
F24	+	–	–	–	–	–	–	–
F29	+	+	–	–	–	–	–	–
F33	+	–	–	–	–	–	–	–
M21	–	–	–	–	–	–	–	+
M31	–	–	–	–	–	–	–	+
M33	+	–	–	–	–	–	–	–
A70	–	–	–	–	+	–	–	–

### qPCR

Of the total impala samples, 7.2% (n = 10/138, 95% CI: 4.0 - 12.8) individuals tested positive for *T. gondii* DNA in at least one tissue. Of these, only two samples [impala F18 (brain) and impala F29 (tongue)] were strong qPCR-positives (Ct values around 25) (Tables 1 and S2 Table). Impala N49 showed weak positivity (42 > Ct values > 25) for three tissues (heart, triceps, and diaphragm), and was the only individual with multiple qPCR-positive tissues (Tables 1 and S2 Table). Each of the other nine impalas only had one positive tissue (Table 1). Of the seven different tissue samples tested per individual impala, the only tissues that contained *T. gondii* DNA were tongue, heart, brain, triceps brachii, and diaphragm (Table 1). No *T. gondii* DNA was found in the liver or spleen of the tested samples (Table 1).

### Comparison between MAT and qPCR

Only three impala (N59, F18, F29) tested both seropositive (MAT) and qPCR-positive (Table 1).

### Genotyping

**Fig 2:**
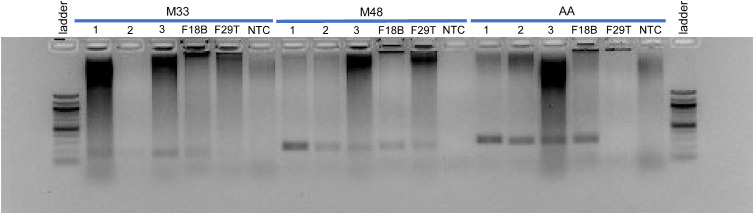
Agarose gel image of some of the microsatellite markers that were amplified by simplex. A few of the microsatellite markers were repeated by performing simplex PCR rather than multiplex PCR results to improve the fragment analysis for the two strong qPCR-positive samples: F18B and F29T. A 100 bp ladder was used for size reference. Known *T. gondii* strains were used as controls: (1) FOU Africa 1 strain, (2) ME49 Type II strain, (3) NED Type III strain. A non-template control (NTC) was run for all markers. Marker M33 has an expected amplicon size of 165–173 bp, marker M48 has an expected amplicon size of 209–243 bp, and marker AA has an expected amplicon size of 251–332 bp [17].

**Fig 3:**
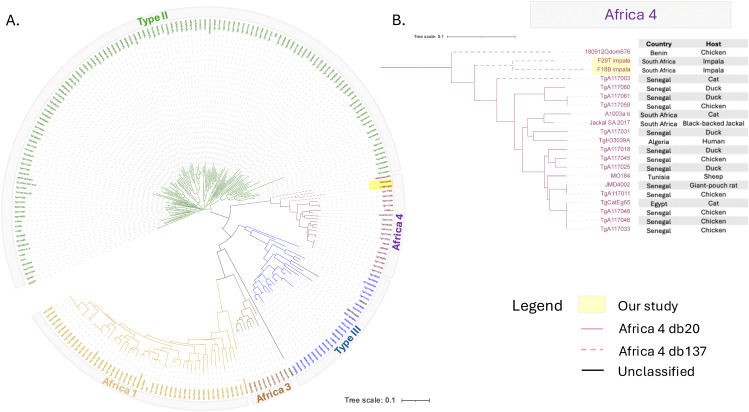
Neighbour-joining tree of *T. gondii* genotypes from various countries, constructed from calculated Bruvo distances using 15 microsatellite markers. In addition to the two strains obtained in the present study, this tree included all African strains previously characterized in the literature using the 15 MS panel, representing 235 strains after exclusion of clonal duplicates within each study country (S3 Table). **(A)** The tree shows the clustering of the main *T. gondii* lineages in Africa. The two Africa 4 genotypes identified in this current study are highlighted in yellow. **(B)** The Africa 4 cluster of some of the Africa 4 genotypes that have been identified in various African countries. The two distinct RFLP sub-lineages, ToxoDB#20 and ToxoDB#137 are indicated by solid branches (Africa 4 db20) and dotted branches (Africa 4 db137), respectively. The two Africa 4 genotypes identified in this study belong to the sub-lineage ToxoDB#137.

The *T. gondii* DNA, present in these two samples, F18B_impala (brain sample) and F29T_impala (tongue sample), was successfully genotyped by fragment analysis of a 15 MS multiplex panel (Table 2). A few markers were repeated using simplex PCR (Fig 2) to confirm the alleles for those markers that initially struggled to amplify as part of the multiplex, and thus have a complete genotype (Table 2). These two strains were characterised as belonging to the Africa 4 lineage by comparing the alleles of the eight lineage typing markers with Africa 4 strains previously described in the literature (Table 2 and Fig 3). The Africa 4 strains from the impala clustered with Africa 4 strains from several African countries (Algeria, Benin, Egypt, Senegal, Tunisia and South Africa) (Fig 3A and S3 Table). Africa 4 lineage comprises two distinct RFLP sub-lineages, ToxoDB#20 and ToxoDB#137, which can be discriminated using the MS marker TUB2, yielding amplicon sizes of 291 bp and 293 bp, respectively (Table 2) [16,27,32]. The two genotyped strains (F18B and F29T) therefore, belonged to the ToxoDB#137 sub-lineage (Table 2 and Fig 3B).

**Table 2:** Genotyping results of *T. gondii* identified in the brain (F18B_impala) and tongue (F29T_impala) of two impala from the TPNR. Toxoplasma reference DNA (FOU, ME49, and NED strains) was also genotyped and used as controls. Previously genotyped Africa 4 strains (TgEgCat65, TgA117003, P676, Mo184, Jackal SA 2017, A1003a and b, H1008 and H1009) are provided for comparison.

ID	TUB2	W35	TgM-A	B18	B17	M33	IV.1	XI.1	M48	M102	N60	N82	AA	N61	N83	Type	Reference (Geographical origin)
FOU	291	248	205	160	342	165	274	354	227	166	147	111	281	89	306	**Africa 1**	**[**3,33**] (France)**
ME49	289	242	207	158	336	169	274	356	215	174	142	111	265	91	310	**Type II**	**[33]** **(USA)**
NED	289	242	205	160	336	165	278	356	209	190	147	111	267	91	312	**Type III**	**[34]** **(France)**
F18B impala	293	242	203	156	336	165	274	354	223	176	132	107	275	95	320	**Africa 4 (ToxoDB#137)**	**This study (South Africa)**
F29T impala	293	242	203	156	336	165	274	354	225	176	134	107	265	97	308	**Africa 4 (ToxoDB#137)**	**This study (South Africa)**
TgEgCat 65	291	242	203	156	336	165	274	354	223	174	130	109	303	99	310	**Africa 4 (ToxoDB#20)**	**[**35,36**] (Egypt)**
TgA117003	293	242	203	156	336	165	274	354	217	174	130	109	281	101	306	**Africa 4 (ToxoDB#137)**	**[35]** **(Senegal)**
P676	293	242	203	156	336	165	274	354	215	174	130^#^	109	291	107	306	**Africa 4 (ToxoDB#137)**	**[37]** **(Benin)**
Mo184	291	242	203	156	336	165	274	354	223	174	130	109	297	99	310	**Africa 4 (ToxoDB#20)**	**[38]** **(Tunisia)**
Jackal_SA_2017	291	242	203	156	336	165	274	354	227	174	130	109	289	NA	310	**Africa 4 (ToxoDB#20)**	[27] **(South Africa)**
A1003a A1003b	291	242	203	156	336	165	274	354	223	174	130	109	287	111	310	**Africa 4 (ToxoDB#20)**	**[39]** **(South Africa)**
H1008	291*	242*	203*	NA	NA	NA	NA	NA	NA	NA	130*	109*	263*	87*	314*	**Africa 4 (ToxoDB#20)**	**[39]** **(South Africa)**
H1009	291*	242*	203*	NA	NA	NA	NA	NA	NA	NA	NA	111*	263*	87*	314*	**Africa 4 (ToxoDB#20)**	**[39]** **(South Africa)**

NA: Not Available

# Corrected allele value from the original article [37] following the guidelines proposed by Joeres [26].

* Allele value obtained after conversion of allelic values between the 8 MS techniques used by Hammond-Aryee [39] and 15 MS [17] using the reference strains employed in Hammond-Aryee [39].

## Discussion

One of the main focuses of the current study was to estimate *T. gondii* seroprevalence in the impala population from the TPNR South Africa, and to determine if specific tissues were predilection sites for infection, to evaluate its potential zoonotic risk and public health impact associated with the consumption of raw (or undercooked) meat of this type of game. A second objective was to assess the genetic diversity of the infecting strains in impala, based on the qPCR-positive impala organs, and to compare it with the known genetic diversity in South Africa and more widely on the African continent.

Results from the MAT showed a seropositivity of 8.7% (n = 12/138, 95% CI: 5.0 - 14.6). The seroprevalence obtained was similar to that previously reported by Bokaba, Dermauw [7]in impala samples from KNP (5.2% using LAT). The difference in seroprevalence values could perhaps be explained by the use of different serological tests. In a study by Dubey, Thulliez [40] that used 1,000 naturally exposed sows, the sensitivity and specificity of MAT were reported to be 82.9% and 90.3%, respectively. While the sensitivity and specificity of LAT were determined as 45.9% and 96.9%, respectively. Thus, MAT has in general, a higher sensitivity than LAT and would be more accurate to determine true positives [41]. MAT detects IgG antibodies, whereas LAT detects both IgG and IgM antibodies [5]. The epidemiology of *T. gondii* is such that a wide variety of factors, including climate, host diversity and environment, play a role in both transmission patterns and circulation intensity, meaning that prevalence rates can vary within a species depending on the region [16]. An example is the way in which impala graze and browse depending on the season, and they might therefore ingest fewer oocysts from the environment than other typical grazing species. Felids (definitive hosts) shed oocysts in their faeces and contaminate the environment, and because of this, they play a central part in the epidemiology and transmission of *T. gondii* [42,43]. Oocysts can be distributed through various means, such as water, grass and harvested feeds, and thus can be ingested by intermediate hosts and cause infection [43]. This dynamic nature of *T. gondii* seropositivity necessitates further research within different areas based on the epidemiological drivers of infection, to more accurately describe the range of infection in impala.

Since one of the sources of *T. gondii* infection in humans is through ingestion of tissue cysts in undercooked meat [43], emphasis should be placed on determining *T. gondii* prevalence within all wildlife species that are regularly consumed as game meat. Previously, *T. gondii* cysts have most often been found in metabolically active tissues as well as specific organs (brain tissue in birds [11], striated muscle and cardiac muscles, tongue, diaphragm, spleen, liver, kidneys, lungs and brain in captive tigers [13] and brain, heart, and tongue in rodents [12]). In this study, *T. gondii* DNA was detected in at least one tissue of 10 impala out of the total of 138 individuals. However, only two impala samples, F18 (brain) and F29 (tongue), were strong qPCR-positives (Ct values around 25). This means that a high concentration of *T. gondii* DNA was detected in the sub-samples from these two impala. It is also important to note that the qPCR assay was not used for quantification purposes but only for qualitative and detection purposes. This is a limitation of the study as the assay was not optimised and validated for quantification.

In our study, *T. gondii* DNA was most frequently found in the brain and triceps brachii in impala, which is in agreement with pre-existing data on the predilection sites in mammals and birds [11–13]. However, future research on a larger sample size might be necessary to confirm these sites for impala and other wild species. The importance of these organs in terms of human consumption should not be overlooked, as not all organs are equally consumed on a regular basis, particularly in South Africa.

The serological and molecular results in our study did not always correlate with each other (Table 1), as already shown in previous studies in West African rodents [44,45]. In other words, a seropositive impala may not have tested positive on the qPCR, and *vice versa*. The discrepancy between MAT and qPCR could be due to several factors. Firstly, it is important to consider what these different tests are used for in practice. Serology is used to determine past exposure to the parasite or aid in the diagnosis of infection [46]. In contrast, qPCR detects the presence of tissue cysts (parasite DNA), qPCR cannot provide information on the viability of the parasite or the level of infection [47]. Bioassays are the definitive way of confirming viable *T. gondii* [47].

For animals that are seropositive but negative in qPCR, one explanation could be that we missed the parasite during sample collection for the molecular approach (DNA extraction). Indeed, *T. gondii* molecular detection is known to be heavily dependent on the type of tissue being sampled, as well as the size and location of the sub-sample taken within that tissue. This is because *T. gondii* tissue cysts are not macroscopically visible and not uniformly distributed throughout the tissues [48]. In this study, selected tissues were sampled, and in each case, only a small piece of tissue was collected and used for DNA extraction (50 mg). Thus, false negative qPCR results could be possible. Another reason for animals that are seropositive but also qPCR negative, could be cross-reaction of the antibodies, meaning that another antibody could potentially recognize the *T. gondii* antigen due to structural similarities to its own specific antigen, and this could lead to a false seropositive result [44,49]. Serological cross-reactions between the *T. gondii* antigen used in MAT serology and antibodies against cystogenic coccidia such, as *Hammondia hammondi*, *Hammondia heydorni* and *Neospora caninum*, as summarised by Gondim, Mineo [50], could therefore occur.

For animals that are seronegative but qPCR-positive, one possible explanation is that the qPCR signal represents a false positive resulting from primer cross-reactivity with Hammondia DNA (Hammondia and Toxoplasma are closely related coccidian parasites) [44]. Another possibility could be that these infected individuals harboured very low concentrations of *T. gondii* DNA, resulting in correspondingly low antibody titers that remained undetectable by MAT. Previous studies have shown that antibody titers in infected hosts may correlate with the parasite burden in the infected tissues [48,51]. In some cases, animals with recent infections may test positive for the parasite DNA before seroconversion [44]. Another potential explanation is vertical transmission of *T. gondii* in impalas, resulting in infected offspring that do not develop detectable antibody levels. Although no data are available for this species, this hypothesis has been discussed by Galal, Schares [44] for small mammals. In their study, the authors propose that in Senegal, some congenitally infected small mammals may remain seronegative, drawing on experimental evidence from laboratory mice demonstrating this phenomenon [52]. This mechanism could also be relevant for impalas, particularly since vertical transmission has been documented at high rates in other ruminants, such as sheep [53].

Limited studies have focused on the genetic characterisation of *T. gondii* genotypes circulating in wildlife in South Africa [15,27] and more broadly across the African continent [54]. *T. gondii* DNA has been detected in various animal species from South Africa, such as African wildcat (*Felis lybica*), dog (*Canis lupus familiaris*), Greater kudu (*Tragelaphus strepsiceros*), honey badger (*Mellivora capensis*), Rusty-spotted genets (*Genetta maculata*), and white-tailed mongoose (*Ichneumia albicauda*); however, genotyping of the corresponding strains was not successful [9,27]. Previous studies have determined that it is difficult and not probable to successfully and completely genotype (with all 15 MS markers) *T. gondii* present in low concentrations, meaning samples with a Ct value above 32 (approximately equivalent to less than 1 copy of Toxoplasma per qPCR) [26,27]. This may explain the difficulty in obtaining genotypes in these studies [9,27]. Our results seem to confirm this rule, since the only samples that could be genotyped were those with Ct values below 32. It is important to note that for a few microsatellite markers, we performed simplex PCR, because the fragment scoring revealed that some markers did not perform optimally in the multiplex panel. These simplex PCR products were run on a gel for QC (Fig 2) and then analysed by capillary electrophoresis to determine or confirm the allele sizes for these markers (Table 2).

In the domestic environment, only ten strains from animal origin have previously been described in South Africa [11,39]. These strains corresponded to both the Type II and Africa 4 lineages. The Type II lineage has been described in a kitten (*Felis catus*), two Squirrel monkeys (*Saimiri sciureus*), two marmosets (*Callithrix* spp.) and two other monkeys (undetermined species) [39], which were kept in captivity in the Western Cape province. Type II has also been described in a wild red-eyed dove (*Streptopelia semitorquata*) from the Limpopo province [11]. More recently, the Africa 4 lineage has been reported in a brain sample of a black-backed jackal (*Lupulella mesomelas*) from the Limpopo province [27]. This lineage has also been described previously in two kittens (*Felis catus*) from the Western Cape province (Table 2), although the authors at the time did not name it Africa 4 (the combination of alleles obtained for each of the microsatellite markers corresponded to this lineage) [39]. Interestingly, in South Africa, the same authors [39] were also able to genotype Toxoplasma DNA from human samples (whole blood, ocular fluid and cerebrospinal fluid) opportunistically collected from patients with acute *T. gondii* infection (without any additional information on the immune status of these patients) at the Tygerberg Academic Hospital and Groote Schuur Academic Hospital in Cape Town from January 2013 to August 2014. Of the 11 human samples genotyped, eight were potentially Type II (based on the three MS markers TUB2, W35, TgMA), one was potentially Type III (based on the three MS markers TUB2, W35, TgMA) and two could belonged to the Africa 4 lineage (based on the four MS markers TUB2, W35, TgMA and N60 close to the specific value of 130 for this lineage) (Table 2) [39]. These results are relevant because they demonstrate that most of the lineages circulating in animals in South Africa also circulate in humans, except for of the Type III lineage, which has not yet been described in animals.

The two strains identified in the present study were assigned to the Africa 4 lineage based on the eight lineage typing markers and the N60 fingerprinting marker allele, which was around 130 bp, a size characteristic of this lineage (Table 2 and Fig 3). The genetic tree analysis confirmed their clustering with other strains belonging to the “Africa 4” lineage and clarified their phylogenetic relationships with the main lineages described on the African continent (Fig 3). The genotyping results indicate that the Africa 4 genotype identified in the two impala corresponds to the ToxoDB#137 RFLP variant. This study reports the first description of the Africa 4 ToxoDB#137 variant in South Africa (Table 2 and Fig 3). This variant of Africa 4 has already been described in domestic animals from Ghana, Benin, Senegal and southwestern China [35,37,44,55,56]. However, for further in-depth phylogenetic and evolutionary analysis among the various Africa 4 and other genotypes, sequencing of *T. gondii* strains will be required. The preliminary genetic tree presented in this study showcases the clustering of lineages seen among all African strains genotyped using 15 MS (Fig 3 and S3 Table). The Africa 4 ToxoDB#20 RFLP variant has already been identified in South Africa, in a black-backed jackal of the Limpopo province [27] and in two kittens from the Western Cape province [39,57], as previously mentioned (Table 2). This variant could also correspond to the two human Africa 4 strains described in the work by Hammond-Aryee [39]. This Africa 4 variant has been described in domestic and livestock animals from Tunisia, Senegal, China, Egypt, Ethiopia, Sri Lanka and the United Arab Emirates (UAE) [35,36,38,58–62] and in human patient from Algeria [63]. The Africa 4 lineage found in the impala (ToxoDB#137) in our study and in a black-backed jackal (ToxoDB#20) from the same region (Limpopo), therefore also appears to circulate in humans [35], despite the very different geographical origins and environments (domestic and wild). The Africa 4 lineage with these 2 variants therefore, seems to be well established in South Africa, both in the wild and in the domestic environment. However, we must remain cautious about these conclusions with regard to human genotypes [35], as these are genotyped using only 8 MS markers, including only 3 typing markers, and not 15 MS markers. It will therefore be necessary in the future to confirm these results in patients using 15 MS markers.

Apart from South Africa and more widely the African continent, for which there is little data on the diversity of human *T. gondii* strains [18], the Africa 4 strains have also been described in human patients in Europe [15]. The ToxoDB#137 variant was identified in a patient with congenital infection from Serbia [64]. The ToxoDB#20 variant was described in an immunocompromised patient with post-HSCT (hematopoietic stem cell transplant) reactivation of severe toxoplasmosis from Serbia [65] as well as in a patient with congenital toxoplasmosis from Bulgaria [64]. The clinical presentation of this lineage in humans remains largely uncharacterized and warrants further investigation. More broadly, clinical outcomes associated with the diverse *T. gondii* lineages circulating across the African continent also remain insufficiently documented.

In this study, impala were sampled from a wildlife slaughterhouse (TPNR) that processes meat for human consumption, and the presence of *T. gondii* strains (toxoplasmosis) can potentially pose health risks to the public. Apart from its non-virulence in laboratory mice and very low parasite load [37], the phenotype and pathogenicity of the Africa 4 strains will need to be investigated to evaluate the severity of the zoonotic risk for humans, especially on the African continent, where too few studies of this kind have been carried out. It would also be pertinent in future studies to investigate the pathogenicity of the Africa 4 strains in the wildlife hosts, as in this current study, there was no information available regarding the health status of the sampled impala. Given that one of the main sources of *T. gondii* infection is through ingestion of tissue cysts in undercooked meat [43], the consumption of infected meat originating from wild animals may contribute to the emergence of potentially more severe toxoplasmosis linked to the parasite’s wild cycle in South Africa, as has been observed in South America [54,66]. In French Guiana, a sylvatic cycle of *T. gondii* has been identified, genetically distinct from the domestic cycle, and associated with increased virulence in humans, leading to severe or even fatal toxoplasmosis in immunocompetent individuals in the absence of treatment [32,66,67]. The current study is the first documented case of the Africa 4 strain detected in the impala antelope in South Africa. This demonstrates that *T. gondii* is circulating in a species that is popular game meat, and this has implications for human health as *T. gondii* can cause toxoplasmosis in immunocompromised individuals. It is also important for animal health as impala are very abundant in the Greater Kruger and a common source of prey for predators. Infected impala may potentially act as a reservoir, facilitating transmission to carnivores through the ingestion of tissue cysts. Future research should therefore prioritize assessing the prevalence of *T. gondii* and characterizing circulating strains in wild species that are frequently hunted and consumed as bushmeat by humans and other animal carnivores.

It is important to note that the game meat industry in South Africa is growing, not only in the rural setting but also for tourism [4]. An effort should be made to include different areas of South Africa in future studies, since different human populations might be at greater risk of infection based on overall health or socio-economic status. Only samples from the Timbavati abattoir (Fig 1) were used in this study, and two strong positive individuals were detected, which indicates the need to perform further research in other wildlife abattoirs to better clarify the risk of *T. gondii* transmission to people consuming game meat from different species. Consumption of these infected tissues, especially undercooked, potentially poses an infection risk. One method to reduce the risk of infection through ingestion of game meat is by ensuring the meat is cooked thoroughly at a temperature of at least 66°C, or by freezing the meat to at least -12°C for 3 days, as both of these methods are sufficient in killing the parasite within tissue cysts [42].

The understanding of local dietary habits, the prevalence of *T. gondii* in wildlife and the phenotype of the strains circulating in these wild-domestic interfaces could thus contribute to improving public health measures and assessing the real risk of infection and transmission between animals and humans. More broadly, it is essential to enhance our understanding of the sylvatic cycle of *T. gondii*, which remains largely understudied in Africa despite the presence of a substantial wildlife reservoir within relatively well-preserved natural habitats [27,54].

## Supporting information

S1 Table Results of modified agglutination test (MAT) to determine seropositivity, as well as the dilution to which seropositivity was detected. The cut-off for seropositivity for MAT is 1/20.
(DOCX)

S2 Table qPCR results for each of the tissue samples collected per individual impala carcass. The qPCR detected *T. gondii* DNA within the impala tissue samples. Ct values below 25 indicated a strong positive sample, whereas Ct values above 25 indicated weak positive samples.
(DOCX)

S3 Table *T. gondii* genotypes identified in various African countries and in the current study, based on the use of 15 microsatellite markers. Three strains (FOU, ME49 and NED) served as known reference strains in the current study. These genotypes were used to construct a neighbour-joining tree for the current study.
(XLSX)

S1 Raw images Fig 2. Agarose gel image of some of the microsatellite markers that were amplified by simplex. A few of the microsatellite markers were repeated by performing simplex PCR rather than multiplex PCR results to improve the fragment analysis for the two strong qPCR positive samples: F18B and F29T. A 100 bp ladder was used for size reference. Known *T. gondii* strains were used as controls: (1) FOU Africa 1 strain, (2) ME49 Type II strain, (3) NED Type III strain. A non-template control (NTC) was run for all markers. Marker M33 has an expected amplicon size of 165–173 bp, marker M48 has an expected amplicon size of 209–243 bp, and marker AA has an expected amplicon size of 251–332 bp [17].
(PDF)
