# Supplementary material for: Molecular epidemiology of Toxoplasma gondii in impala (Aepyceros melampus) from the Greater Kruger in South Africa: Detection of the Africa 4 lineage
Source: PLoS Negl Trop Dis. 2026 Jul 9;20(7):e0014475. doi: 10.1371/journal.pntd.0014475 (PMC13379095; doi:10.1371/journal.pntd.0014475)
Supplement: S1 Table — The cut-off for seropositivity for MAT is 1/20. (DOCX) [file pntd.0014475.s001.docx]

**S1 Table. Results of modified agglutination test (MAT) to determine seropositivity, as well as the dilution to which seropositivity was detected.**The cut-off for seropositivity for MAT is 1/20.

|  | **MAT result** | |
| --- | --- | --- |
| **Sample number*** | **Positive or Negative** | **Dilution of positivity** |
| **N1** | **-** | **-** |
| **N2** | **-** | **-** |
| **N3** | **-** | **-** |
| **N4** | **-** | **-** |
| **N5** | **+** | **1/40** |
| **N8** | **-** | **-** |
| **N9** | **-** | **-** |
| **N10** | **-** | **-** |
| **N11** | **-** | **-** |
| **N12** | **-** | **-** |
| **N29** | **-** | **-** |
| **N30** | **-** | **-** |
| **N31** | **-** | **-** |
| **N32** | **-** | **-** |
| **N33** | **-** | **-** |
| **N34** | **-** | **-** |
| **N35** | **-** | **-** |
| **N36** | **+** | **1/100** |
| **N38** | **-** | **-** |
| **N39** | **-** | **-** |
| **N40** | **-** | **-** |
| **N41** | **-** | **-** |
| **N42** | **-** | **-** |
| **N48** | **-** | **-** |
| **N49** | **-** | **-** |
| **N50** | **-** | **-** |
| **N51** | **-** | **-** |
| **N52** | **-** | **-** |
| **N53** | **-** | **-** |
| **N54** | **-** | **-** |
| **N55** | **-** | **-** |
| **N56** | **-** | **-** |
| **N57** | **-** | **-** |
| **N58** | **-** | **-** |
| **N59** | **+** | **1/40** |
| **N60** | **-** | **-** |
| **N61** | **-** | **-** |
| **N64** | **-** | **-** |
| **N65** | **-** | **-** |
| **N66** | **+** | **1/40** |
| **N67** | **-** | **-** |
| **N68** | **-** | **-** |
| **N76** | **-** | **-** |
| **N77** | **-** | **-** |
| **N78** | **-** | **-** |
| **N79** | **-** | **-** |
| **N80** | **-** | **-** |
| **N81** | **-** | **-** |
| **N82** | **-** | **-** |
| **N85** | **-** | **-** |
| **N86** | **-** | **-** |
| **N87** | **-** | **-** |
| **D1** | **-** | **-** |
| **D2** | **-** | **-** |
| **D3** | **+** | **1/40** |
| **D4** | **-** | **-** |
| **D5** | **-** | **-** |
| **D6** | **+** | **1/100** |
| **D7** | **-** | **-** |
| **D8** | **-** | **-** |
| **D9** | **-** | **-** |
| **D10** | **-** | **-** |
| **D11** | **-** | **-** |
| **D12** | **-** | **-** |
| **J11** | **-** | **-** |
| **J12** | **-** | **-** |
| **J23** | **-** | **-** |
| **J24** | **-** | **-** |
| **J25** | **-** | **-** |
| **F1** | **-** | **-** |
| **F2** | **-** | **-** |
| **F4** | **-** | **-** |
| **F5** | **-** | **-** |
| **F6** | **-** | **-** |
| **F7** | **-** | **-** |
| **F8** | **-** | **-** |
| **F12** | **-** | **-** |
| **F13** | **-** | **-** |
| **F14** | **-** | **-** |
| **F15** | **+** | **1/40** |
| **F16** | **-** | **-** |
| **F18** | **+** | **1/800** |
| **F19** | **-** | **-** |
| **F20** | **-** | **-** |
| **F21** | **-** | **-** |
| **F22** | **-** | **-** |
| **F23** | **-** | **-** |
| **F24** | **+** | **1/400** |
| **F25** | **-** | **-** |
| **F26** | **-** | **-** |
| **F27** | **-** | **-** |
| **F28** | **-** | **-** |
| **F29** | **+** | **1/800** |
| **F31** | **-** | **-** |
| **F32** | **-** | **-** |
| **F33** | **+** | **1/40** |
| **F35** | **-** | **-** |
| **M1** | **-** | **-** |
| **M2** | **-** | **-** |
| **M3** | **-** | **-** |
| **M19** | **-** | **-** |
| **M20** | **-** | **-** |
| **M21** | **-** | **-** |
| **M26** | **-** | **-** |
| **M27** | **-** | **-** |
| **M29** | **-** | **-** |
| **M30** | **-** | **-** |
| **M31** | **-** | **-** |
| **M32** | **-** | **-** |
| **M33** | **+** | **1/40** |
| **M49** | **-** | **-** |
| **M69** | **-** | **-** |
| **M70** | **-** | **-** |
| **M71** | **-** | **-** |
| **M72** | **-** | **-** |
| **M74** | **-** | **-** |
| **M75** | **-** | **-** |
| **M76** | **-** | **-** |
| **M77** | **-** | **-** |
| **A16** | **-** | **-** |
| **A17** | **-** | **-** |
| **A18** | **-** | **-** |
| **A19** | **-** | **-** |
| **A20** | **-** | **-** |
| **A21** | **-** | **-** |
| **A22** | **-** | **-** |
| **A23** | **-** | **-** |
| **A30** | **-** | **-** |
| **A31** | **-** | **-** |
| **A32** | **-** | **-** |
| **A35** | **-** | **-** |
| **A37** | **-** | **-** |
| **A38** | **-** | **-** |
| **A40** | **-** | **-** |
| **A41** | **-** | **-** |
| **A47** | **-** | **-** |
| **A70** | **-** | **-** |
| **A72** | **-** | **-** |

*The letter of each sample number correlates to the month in which the samples were collected:

·         N – November 2023

·         D – December 2023

·         J – January 2024

·         F – February 2024

·         M – March 2024

·         A – April 2024

The total number of animals was calculated based on both the monthly slaughter count and the samples taken. Since the Timbavati abattoir is an official institution, meat inspection guidelines often resulted in some carcasses being condemned and were thus not sampled for the purposes of this study.
