# Supplementary material for: Molecular epidemiology of Toxoplasma gondii in impala (Aepyceros melampus) from the Greater Kruger in South Africa: Detection of the Africa 4 lineage
Source: PLoS Negl Trop Dis. 2026 Jul 9;20(7):e0014475. doi: 10.1371/journal.pntd.0014475 (PMC13379095; doi:10.1371/journal.pntd.0014475)
Supplement: S2 Table — The qPCR detected T. gondii DNA within the impala tissue samples. Ct values below 25 indicated a strong positive sample, whereas Ct values above 25 indicated weak positive samples. (DOCX) [file pntd.0014475.s002.docx]

**S2 Table. qPCR results for each of the tissue samples collected per individual impala carcass.** The qPCR detected *T. gondii* DNA within the impala tissue samples. Ct values below 25 indicated a strong positive sample, whereas Ct values above 25 indicated weak positive samples.

|  | **qPCR: Positive (Ct value provided) or Negative** | | | | | | |
| --- | --- | --- | --- | --- | --- | --- | --- |
| **Sample number** | **Tongue** | **Heart** | **Brain** | **Liver** | **Triceps** | **Diaphragm** | **Spleen** |
| **N1** | **-** | **-** | **-** | **-** | **-** | **-** | **-** |
| **N2** | **-** | **-** | **-** | **-** | **-** | **-** | **-** |
| **N3** | **-** | **-** | **-** | **-** | **-** | **-** | **-** |
| **N4** | **-** | **-** | **-** | **-** | **-** | **-** | **-** |
| **N5** | **-** | **-** | **-** | **-** | **-** | **-** | **-** |
| **N8** | **-** | **-** | **-** | **-** | **-** | **-** | **-** |
| **N9** | **-** | **-** | **-** | **-** | **-** | **-** | **-** |
| **N10** | **-** | **-** | **-** | **-** | **Weak + (41.65)** | **-** | **-** |
| **N11** | **-** | **-** | **-** | **-** | **-** | **-** | **-** |
| **N12** | **-** | **-** | **-** | **-** | **-** | **-** | **-** |
| **N29** | **-** | **-** | **-** | **-** | **-** | **-** | **-** |
| **N30** | **-** | **-** | **-** | **-** | **-** | **-** | **-** |
| **N31** | **-** | **-** | **-** | **-** | **-** | **-** | **-** |
| **N32** | **-** | **-** | **-** | **-** | **-** | **-** | **-** |
| **N33** | **-** | **-** | **-** | **-** | **-** | **-** | **-** |
| **N34** | **-** | **-** | **-** | **-** | **-** | **-** | **-** |
| **N35** | **-** | **-** | **-** | **-** | **-** | **-** | **-** |
| **N36** | **-** | **-** | **-** | **-** | **-** | **-** | **-** |
| **N38** | **-** | **-** | **-** | **-** | **-** | **-** | **-** |
| **N39** | **-** | **-** | **-** | **-** | **-** | **-** | **-** |
| **N40** | **-** | **-** | **-** | **-** | **-** | **-** | **-** |
| **N41** | **-** | **-** | **-** | **-** | **-** | **-** | **-** |
| **N42** | **-** | **-** | **-** | **-** | **-** | **-** | **-** |
| **N48** | **-** | **-** | **-** | **-** | **-** | **-** | **-** |
| **N49** | **-** | **Weak + (35.90)** | **-** | **-** | **Weak + (41.94)** | **Weak + (35.26)** | **-** |
| **N50** | **-** | **-** | **-** | **-** | **-** | **Weak + (37.32)** | **-** |
| **N51** | **-** | **-** | **-** | **-** | **-** | **-** | **-** |
| **N52** | **-** | **-** | **-** | **-** | **-** | **-** | **-** |
| **N53** | **-** | **-** | **-** | **-** | **-** | **-** | **-** |
| **N54** | **-** | **-** | **-** | **-** | **-** | **-** | **-** |
| **N55** | **-** | **-** | **-** | **-** | **-** | **-** | **-** |
| **N56** | **-** | **-** | **-** | **-** | **-** | **-** | **-** |
| **N57** | **-** | **-** | **-** | **-** | **-** | **-** | **-** |
| **N58** | **-** | **-** | **-** | **-** | **-** | **-** | **-** |
| **N59** | **-** | **-** | **Weak + (39.68)** | **-** | **-** | **-** | **-** |
| **N60** | **-** | **-** | **-** | **-** | **-** | **-** | **-** |
| **N61** | **-** | **-** | **-** | **-** | **Weak + (37.52)** | **-** | **-** |
| **N64** | **-** | **-** | **-** | **-** | **-** | **-** | **-** |
| **N65** | **-** | **-** | **-** | **-** | **-** | **-** | **-** |
| **N66** | **-** | **-** | **-** | **-** | **-** | **-** | **-** |
| **N67** | **-** | **-** | **-** | **-** | **-** | **-** | **-** |
| **N68** | **-** | **-** | **-** | **-** | **-** | **-** | **-** |
| **N76** | **-** | **-** | **-** | **-** | **-** | **-** | **-** |
| **N77** | **-** | **-** | **-** | **-** | **-** | **-** | **-** |
| **N78** | **-** | **-** | **-** | **-** | **-** | **-** | **-** |
| **N79** | **-** | **-** | **-** | **-** | **-** | **-** | **-** |
| **N80** | **-** | **-** | **-** | **-** | **-** | **-** | **-** |
| **N81** | **-** | **-** | **-** | **-** | **-** | **-** | **-** |
| **N82** | **-** | **-** | **-** | **-** | **-** | **-** | **-** |
| **N85** | **-** | **-** | **-** | **-** | **-** | **-** | **-** |
| **N86** | **-** | **-** | **-** | **-** | **-** | **-** | **-** |
| **N87** | **-** | **-** | **-** | **-** | **-** | **-** | **-** |
| **D1** | **-** | **-** | **-** | **-** | **-** | **-** | **-** |
| **D2** | **-** | **-** | **-** | **-** | **-** | **-** | **-** |
| **D3** | **-** | **-** | **-** | **-** | **-** | **-** | **-** |
| **D4** | **-** | **-** | **-** | **-** | **-** | **-** | **-** |
| **D5** | **-** | **-** | **-** | **-** | **-** | **-** | **-** |
| **D6** | **-** | **-** | **-** | **-** | **-** | **-** | **-** |
| **D7** | **-** | **-** | **-** | **-** | **-** | **-** | **-** |
| **D8** | **-** | **-** | **-** | **-** | **-** | **-** | **-** |
| **D9** | **-** | **-** | **-** | **-** | **-** | **-** | **-** |
| **D10** | **-** | **-** | **-** | **-** | **-** | **-** | **-** |
| **D11** | **-** | **-** | **-** | **-** | **-** | **-** | **-** |
| **D12** | **-** | **-** | **-** | **-** | **-** | **-** | **-** |
| **J11** | **-** | **-** | **-** | **-** | **-** | **-** | **-** |
| **J12** | **-** | **-** | **-** | **-** | **-** | **-** | **-** |
| **J23** | **-** | **-** | **-** | **-** | **-** | **-** | **-** |
| **J24** | **-** | **-** | **-** | **-** | **-** | **-** | **-** |
| **J25** | **-** | **-** | **-** | **-** | **-** | **-** | **-** |
| **F1** | **-** | **-** | **-** | **-** | **-** | **-** | **-** |
| **F2** | **-** | **-** | **-** | **-** | **-** | **-** | **-** |
| **F4** | **-** | **-** | **-** | **-** | **-** | **-** | **-** |
| **F5** | **-** | **-** | **-** | **-** | **-** | **-** | **-** |
| **F6** | **-** | **-** | **-** | **-** | **-** | **-** | **-** |
| **F7** | **-** | **-** | **-** | **-** | **-** | **-** | **-** |
| **F8** | **-** | **-** | **-** | **-** | **-** | **-** | **-** |
| **F12** | **-** | **-** | **-** | **-** | **-** | **-** | **-** |
| **F13** | **-** | **-** | **-** | **-** | **-** | **-** | **-** |
| **F14** | **-** | **-** | **-** | **-** | **-** | **-** | **-** |
| **F15** | **-** | **-** | **-** | **-** | **-** | **-** | **-** |
| **F16** | **-** | **-** | **-** | **-** | **-** | **-** | **-** |
| **F18** | **-** | **-** | **Strong + (25.39)** | **-** | **-** | **-** | **-** |
| **F19** | **-** | **-** | **-** | **-** | **-** | **-** | **-** |
| **F20** | **-** | **-** | **-** | **-** | **-** | **-** | **-** |
| **F21** | **-** | **-** | **-** | **-** | **-** | **-** | **-** |
| **F22** | **-** | **-** | **-** | **-** | **-** | **-** | **-** |
| **F23** | **-** | **-** | **-** | **-** | **-** | **-** | **-** |
| **F24** | **-** | **-** | **-** | **-** | **-** | **-** | **-** |
| **F25** | **-** | **-** | **-** | **-** | **-** | **-** | **-** |
| **F26** | **-** | **-** | **-** | **-** | **-** | **-** | **-** |
| **F27** | **-** | **-** | **-** | **-** | **-** | **-** | **-** |
| **F28** | **-** | **-** | **-** | **-** | **-** | **-** | **-** |
| **F29** | **Strong + (25.21)** | **-** | **-** | **-** | **-** | **-** | **-** |
| **F31** | **-** | **-** | **-** | **-** | **-** | **-** | **-** |
| **F32** | **-** | **-** | **-** | **-** | **-** | **-** | **-** |
| **F33** | **-** | **-** | **-** | **-** | **-** | **-** | **-** |
| **F35** | **-** | **-** | **-** | **-** | **-** | **-** | **-** |
| **M1** | **-** | **-** | **-** | **-** | **-** | **-** | **-** |
| **M2** | **-** | **-** | **-** | **-** | **-** | **-** | **-** |
| **M3** | **-** | **-** | **-** | **-** | **-** | **-** | **-** |
| **M19** | **-** | **-** | **-** | **-** | **-** | **-** | **-** |
| **M20** | **-** | **-** | **-** | **-** | **-** | **-** | **-** |
| **M21** | **-** | **-** | **Weak + (37.56)** | **-** | **-** | **-** | **-** |
| **M26** | **-** | **-** | **-** | **-** | **-** | **-** | **-** |
| **M27** | **-** | **-** | **-** | **-** | **-** | **-** | **-** |
| **M29** | **-** | **-** | **-** | **-** | **-** | **-** | **-** |
| **M30** | **-** | **-** | **-** | **-** | **-** | **-** | **-** |
| **M31** | **-** | **-** | **Weak + (41.50)** | **-** | **-** | **-** | **-** |
| **M32** | **-** | **-** | **-** | **-** | **-** | **-** | **-** |
| **M33** | **-** | **-** | **-** | **-** | **-** | **-** | **-** |
| **M49** | **-** | **-** | **-** | **-** | **-** | **-** | **-** |
| **M69** | **-** | **-** | **-** | **-** | **-** | **-** | **-** |
| **M70** | **-** | **-** | **-** | **-** | **-** | **-** | **-** |
| **M71** | **-** | **-** | **-** | **-** | **-** | **-** | **-** |
| **M72** | **-** | **-** | **-** | **-** | **-** | **-** | **-** |
| **M74** | **-** | **-** | **-** | **-** | **-** | **-** | **-** |
| **M75** | **-** | **-** | **-** | **-** | **-** | **-** | **-** |
| **M76** | **-** | **-** | **-** | **-** | **-** | **-** | **-** |
| **M77** | **-** | **-** | **-** | **-** | **-** | **-** | **-** |
| **A16** | **-** | **-** | **-** | **-** | **-** | **-** | **-** |
| **A17** | **-** | **-** | **-** | **-** | **-** | **-** | **-** |
| **A18** | **-** | **-** | **-** | **-** | **-** | **-** | **-** |
| **A19** | **-** | **-** | **-** | **-** | **-** | **-** | **-** |
| **A20** | **-** | **-** | **-** | **-** | **-** | **-** | **-** |
| **A21** | **-** | **-** | **-** | **-** | **-** | **-** | **-** |
| **A22** | **-** | **-** | **-** | **-** | **-** | **-** | **-** |
| **A23** | **-** | **-** | **-** | **-** | **-** | **-** | **-** |
| **A30** | **-** | **-** | **-** | **-** | **-** | **-** | **-** |
| **A31** | **-** | **-** | **-** | **-** | **-** | **-** | **-** |
| **A32** | **-** | **-** | **-** | **-** | **-** | **-** | **-** |
| **A35** | **-** | **-** | **-** | **-** | **-** | **-** | **-** |
| **A37** | **-** | **-** | **-** | **-** | **-** | **-** | **-** |
| **A38** | **-** | **-** | **-** | **-** | **-** | **-** | **-** |
| **A40** | **-** | **-** | **-** | **-** | **-** | **-** | **-** |
| **A41** | **-** | **-** | **-** | **-** | **-** | **-** | **-** |
| **A47** | **-** | **-** | **-** | **-** | **-** | **-** | **-** |
| **A70** | **-** | **-** | **-** | **-** | **Weak + (41.56)** | **-** | **-** |
| **A72** | **-** | **-** | **-** | **-** | **-** | **-** | **-** |
