# Supplementary material for: Molecular epidemiology of Toxoplasma gondii in impala (Aepyceros melampus) from the Greater Kruger in South Africa: Detection of the Africa 4 lineage
Source: PLoS Negl Trop Dis. 2026 Jul 9;20(7):e0014475. doi: 10.1371/journal.pntd.0014475 (PMC13379095; doi:10.1371/journal.pntd.0014475)
Supplement: S1 Raw images — A few of the microsatellite markers were repeated by performing simplex PCR rather than multiplex PCR results to improve the fragment analysis for the two strong qPCR positive samples: F18B and F29T. A 100 bp ladder was used for size reference. Known T. gondii strains were used as controls: (1) FOU Africa 1 strain, (2) ME49 Type II strain, (3) NED Type III strain. A non-template control (NTC) was run for all markers. Marker M33 has an expected amplicon size of 165–173 bp, marker M48 has an expected amplicon size of 209–243 bp, and marker AA has an expected amplicon size of 251–332 bp [17]. (PDF) [file pntd.0014475.s004.pdf]

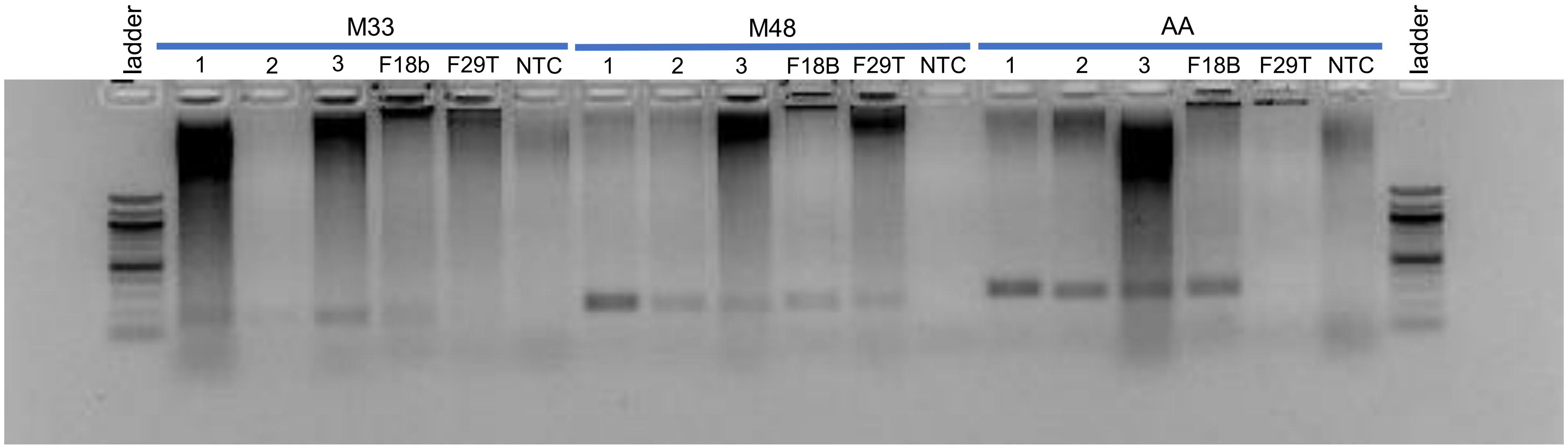


**Fig 2. Agarose gel image of some of the microsatellite markers that were amplified by simplex.** A few of the microsatellite markers were repeated by performing simplex PCR rather than multiplex PCR results to improve the fragment analysis for the two strong qPCR positive samples: F18B and F29T. A 100 bp ladder was used for size reference. Known *T. gondii* strains were used as controls: (1) FOU Africa 1 strain, (2) ME49 Type II strain, (3) NED Type III strain. A non-template control (NTC) was run for all markers. Marker M33 has an expected amplicon size of 165–173 bp, marker M48 has an expected amplicon size of 209–243 bp, and marker AA has an expected amplicon size of 251–332 bp [17].
